# Reversible Oxidative Modifications in Myoglobin and Functional Implications

**DOI:** 10.3390/antiox9060549

**Published:** 2020-06-24

**Authors:** Mark H. Mannino, Rishi S. Patel, Amanda M. Eccardt, Blythe E. Janowiak, David C. Wood, Fahu He, Jonathan S. Fisher

**Affiliations:** 1 Department of Biology, Saint Louis University, St. Louis, MO 63103, USA; mark.mannino@utsouthwestern.edu (M.H.M.); patel559@purdue.edu (R.S.P.); Amanda.Eccardt@pfizer.com (A.M.E.); blythe.janowiakmulligan@slu.edu (B.E.J.); 2 Department of Biochemistry and Molecular Biology, Saint Louis University, St. Louis, MO 63104, USA; david.wood@health.slu.edu; 3 Department of Chemistry, Saint Louis University, St. Louis, MO 63103, USA; fahu.he@slu.edu

**Keywords:** myoglobin, dityrosine, ditryptophan, peroxidase, protein aggregation

## Abstract

Myoglobin (Mb), an oxygen-binding heme protein highly expressed in heart and skeletal muscle, has been shown to undergo oxidative modifications on both an inter- and intramolecular level when exposed to hydrogen peroxide (H_2_O_2_) in vitro. Here, we show that exposure to H_2_O_2_ increases the peroxidase activity of Mb. Reaction of Mb with H_2_O_2_ causes covalent binding of heme to the Mb protein (Mb-X), corresponding to an increase in peroxidase activity when ascorbic acid is the reducing co-substrate. Treatment of H_2_O_2_-reacted Mb with ascorbic acid reverses the Mb-X crosslink. Reaction with H_2_O_2_ causes Mb to form dimers, trimers, and larger molecular weight Mb aggregates, and treatment with ascorbic acid regenerates Mb monomers. Reaction of Mb with H_2_O_2_ causes formation of dityrosine crosslinks, though the labile nature of the crosslinks broken by treatment with ascorbic acid suggests that the reversible aggregation of Mb is mediated by crosslinks other than dityrosine. Disappearance of a peptide containing a tryptophan residue when Mb is treated with H_2_O_2_ and the peptide’s reappearance after subsequent treatment with ascorbic acid suggest that tryptophan side chains might participate in the labile crosslinking. Taken together, these data suggest that while exposure to H_2_O_2_ causes Mb-X formation, increases Mb peroxidase activity, and causes Mb aggregation, these oxidative modifications are reversible by treatment with ascorbic acid. A caveat is that future studies should demonstrate that these and other in vitro findings regarding properties of Mb have relevance in the intracellular milieu, especially in regard to actual concentrations of metMb, H_2_O_2_, and ascorbate that would be found in vivo.

## 1. Introduction

One of the major mechanisms of toxicity from reactive oxygen species (ROS) is the direct oxidation of protein side chains [1]. Although some oxidations are reversible, such as oxidation of the cysteine thiol to sulfenic acid, the majority are considered to be irreversible and to promote the destabilization of tertiary structure as well as the eventual loss of protein function [2,3]. One group of proteins that are particularly susceptible to oxidative damage is heme proteins [4,5,6]. This is likely due to the redox activity of the porphyrin-centered iron. When present in the ferric state (III), heme proteins are prone to oxidation by endogenously produced H_2_O_2_, resulting in a highly unstable oxoferryl form, which can then oxidize protein side chains either internally or on another protein [7].

One oxidative modification known to occur in myoglobin (Mb), especially under acidic conditions [8], involves the covalent linkage between the heme group and protein side chains [9,10,11]. It has been suggested that Mb cross-linked to heme be referred to as Mb-X to delineate it from the abbreviation for abnormal hemoglobin associated with Thalassemia (Mb-H) and the proposed sites of cross-linking, Mb-H when linked at a histidine and Mb-Y when linked via a tyrosine [8]. This paper will use the Mb-X notation to refer to heme-to-protein covalent bonds. The Mb-X form of Mb has increased NADH oxidase activity [12] and oxidizes low density lipoprotein (LDL), phospholipids, and cholesterol esters more rapidly than native Mb [13]. Mb-X also promotes cell death when taken up by cultured fibroblasts [14]. Mb-X and F2-isoprostanes, peroxidation products of arachidonic acid known to be produced by Mb [15] were increased in the urine of rhabdomyolysis patients [16], suggesting a role of Mb-X in the pathology of rhabdomyolysis.

Another oxidative modification shown to be present in H_2_O_2_-treated Mb is dityrosine. Dityrosine cross-links can be formed both intra- and intermolecularly [17,18] by the o,o’ coupling of two tyrosinyl radicals. Dityrosine cross-links have been viewed as markers of oxidative stress in vivo [19,20,21,22], although there is evidence that they might play a causal role in age-related pathologies such as Alzheimer’s [23] and Parkinson’s disease [24].

Here, we show that reaction of Mb with H_2_O_2_ increases peroxidase activity when ascorbate is the reducing co-substrate, a change that is associated with Mb-X formation. Furthermore, treatment of H_2_O_2_-reacted Mb with ascorbic acid reverses the Mb-X crosslink. We also show that Mb aggregates formed upon reaction of Mb with H_2_O_2_ are broken by subsequent treatment with ascorbic acid. In addition, it appears that Mb dimer reversal is protein catalyzed, as heat and detergent denatured Mb dimers were unable to reverse their cross-links. In summary, we find that oxidative modifications of Mb including formation of Mb-X and Mb aggregates are reversible by treatment with ascorbic acid, suggesting that Mb might serve a novel role of reversing oxidative modifications in proteins.

## 2. Materials and Methods

### 2.1. Reagents

Horse heart myoglobin, 30% H_2_O_2_, 3,3′,5,5′-tetramethylbenzidine (TMB), caffeic acid, resveratrol, N-acetylimidazole, methanol, acetonitrile, ethylenediaminetetraacetic acid (EDTA), dihydrobenzoic acid, and NADH were purchased from Sigma Aldrich Corporation (St. Louis, MO, USA). Ascorbic acid was from ICN Biomedicals Inc. (Aurora, OH, USA). NADPH was from Enzo Life Sciences (Farmingdale, NY, USA). 2-butanone was from Acros Organics/Thermo Fisher Scientific (Waltham, MA, USA). Sequencing grade trypsin was from Roche (Indianapolis, IN, USA).

### 2.2. Metmyoglobin Peroxidase Activity Assays

Metmyoglobin (metMb) solutions were prepared in 50 mM sodium phosphate buffer, pH 7.4. MetMb (111 µM) was incubated with 50–200 µM H_2_O_2_, and aliquots of these solutions were added to a reaction mixture containing 250 µM ascorbic acid or 500 µM TMB. Peroxidase activity was monitored by disappearance of ascorbate (ε_290 nm_ = 2900 M^−1^ cm^−1^) or reduction of TMB (ε_653 nm_ = 39,000 M^−1^ cm^−1^) as we have previously described [25].

### 2.3. Nuclear Magnetic Resonance (NMR) Methods

NMR procedures are based on NMR methods described for analysis of hemoproteins [26,27]. NMR samples were prepared in H_2_O with 10% D_2_O for locking. pH, uncorrected for the isotope effect, was adjusted with dilute HCl or NaOH. Proton NMR spectra were obtained on a Bruker AVANCE III HD 700 spectrometer equipped with a QCI cryoprobe (Bruker Biospin, Rheinstetten, Germany) at 16.44 Tesla at room temperature (298K). One-dimension (1D) ^1^H spectra were acquired with a p3919gp pulse sequence with ~50 ppm spectrum width and 200 ms recycle delay.

### 2.4. Heme Stain

metMb samples were incubated in the presence or absence of H_2_O_2_ and then subjected to sodium dodecyl sulfate polyacrylamide gel electrophoresis (SDS-PAGE). Heme stains were done using an established protocol [28] for assessing heme peroxidase activity in polyacrylamide gels. Staining solutions were made by first dissolving 23 mg TMB in 15 mL methanol, which was then added to 35 mL of 250 mM sodium acetate buffer, pH 5.0. After incubating gels in this staining solution for ~1 h, 180 μL of 30% H_2_O_2_ was added, and reactions were stopped by washing with deionized H_2_O after blue bands corresponding to oxidized TMB appeared. Bands were photo-documented with a LAS-4000 ImageQuant (GE Healthcare Life Sciences, Marlborough, MA, USA) or an iBright FL1000 (Thermo Fisher Scientific, Waltham, MA, USA).

### 2.5. Heme Extraction

Non-covalently bound heme was removed using the acid-butanone method established by Catalano et al. [10]. Mb solutions (treated or untreated) were first cooled on ice for 10 min. Next, concentrated HCl was added to a pH of 1–1.5. After incubating the acidified solutions on ice for another 10 min, a 2:1 volume ratio of chilled 2-butanone was added before mixing and incubating on ice for 5 min. The amount of heme in the organic phase was determined by measuring the absorbance at 398 nm on a Genysis 5 spectrophotometer (Spectronic Instruments Inc., Fitchburg, WI, USA). To obtain Mb covalently linked to heme (Mb-X), the aqueous phase was removed, and the sample was dialyzed against 50 mM sodium phosphate buffer (pH 7.4) overnight to remove contaminant butanone.

### 2.6. Mb-X Peroxidase Activity Assays

The peroxidase activity of Mb-X was followed spectrophotometrically. Reactions were done in 50 mM sodium phosphate buffer containing 200 μM hydrogen peroxide. Reaction progress over time was measured using the following extinction coefficients: ε _312 nm_ = 11,200 M^−1^ cm^−1^ for caffeic acid; ε _304 nm_ = 19,406 M^−1^ cm^−1^ for resveratrol; ε _340 nm_ = 6270 M^−1^ cm^−1^ for NADH and NADPH.

### 2.7. Matrix-Assisted Laser Desorption/Ionization Time-of-Flight Mass Spectrometry (MALDI-TOF MS)

Samples for analysis using MALDI-TOF MS were prepared as described by Shevchenko et al. [29]. Briefly, samples were first run on a pre-cast SDS-PAGE gel. Next, bands were excised and sliced into small pieces using a clean spatula. Gel slices were then shrunken by adding 0.5 mL acetonitrile for 10 min. In-gel digestion was then performed by immersing the dehydrated gel slices in a buffer containing 10 mM NH_4_HCO_3_, 10% acetonitrile, and 13 ng/μL trypsin (modified, sequencing grade). Samples were kept for either 4 h at 37 °C or overnight at room temperature before placing them at −20 °C until MALDI-TOF analysis. Proteolytic digests were added to a plate containing an equal volume of 20 g/L dihydroxybenzoic acid and were allowed to dry. Spectra were then acquired using a Shimadzu Resonance(tm) MALDI-QIT-TOF mass spectrometer (Shimadzu Scientific Instruments, Columbia, MD, USA).

### 2.8. Detection of Dityrosine Using Fluorescence

To detect dityrosine fluorescence using the fluorescence spectra described by Malencik et al. [17], fluorescence was measured at excitation and emission wavelengths of 290 nm and 400 nm, respectively, with a Synergy H1 platereader (Biotek, Winooski, VT, USA).

### 2.9. Western Blot Using Anti-dityrosine Antibody

Mb (2 mg/mL) samples were first reacted with 300 μM H_2_O_2_ for 5, 10, 20, and 40 min. Some of these H_2_O_2_-reacted samples were then treated with 833 μM ascorbic acid for the same duration of time they had been reacted with H_2_O_2_. Samples were detected via Western blotting using an anti-dityrosine antibody (Japan Institute for the Control of Aging, Shizuoka, Japan) and an Alexa™ 488 secondary antibody (with excitation and emission maxima of 490 nm and 525 nm respectively, Invitrogen, Carlsbad, CA, USA)**.** We needed to use the fluorescent secondary antibody, because chemiluminescent detection would be incompatible with the presence of heme; if we were to use enhanced chemiluminescence substrate, bands would appear wherever heme were present (i.e., in the same pattern shown by the in-gel heme peroxidase activity stain using TMB), not just in dityrosine-containing bands.

### 2.10. Tyrosine Acetylation Using N-Acetylimidazole

Mb was acetylated according to a procedure described by Basu et al. [30]. Briefly, Mb was incubated in 50 mM sodium phosphate buffer, pH 7.4, containing a 10:1 molar excess of N-acetylimidazole, and samples were mixed overnight at room temperature. Excess N-acetylimidazole was removed by washes with 50 mM sodium phosphate buffer, pH 7.4, followed by spinning the acetylated samples in G-Sephadex Millipore spin filter columns (molecular weight cutoff = 10 kDa, Millipore, St. Louis, MO, USA).

### 2.11. Statistical Methods

For experiments with time courses, data were analyzed by analysis of variance (ANOVA) with time as a factor. When time course data involved more than two groups, an ANOVA using data from all groups with time as a factor was performed. If this model was significant (*p* < 0.05), post-hoc comparisons were separately made between the control group and each other group using ANOVA with time as a factor. Experiments with end-point data were analyzed by ANOVA with least significant difference (LSD) post-hoc comparisons when appropriate (*p* < 0.05).

## 3. Results

### 3.1. Pre-treatment with H_2_O_2_ Increases Mb Peroxidase Activity

**Figure 1:**
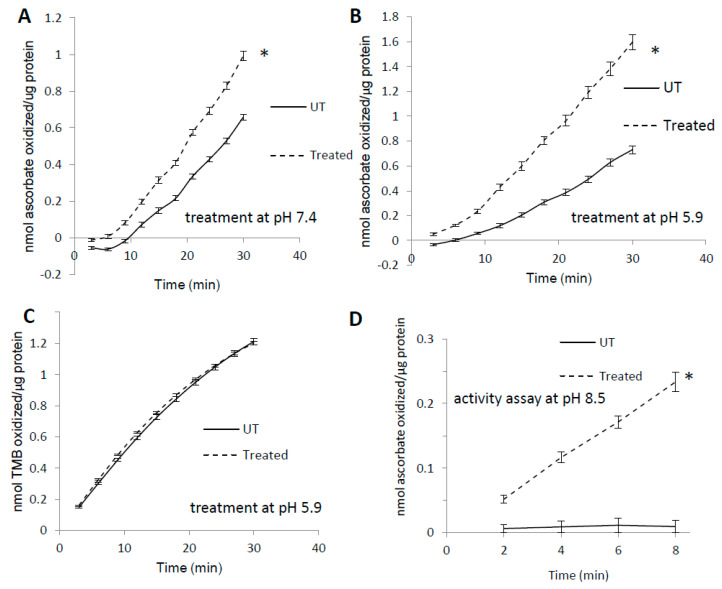
Pre-treatment with H_2_O_2_ increases MetMb peroxidase activity in a substrate-dependent manner. MetMb (111 μM, pH 5.9) was untreated (UT) or was pre-reacted with 50 μM H_2_O_2_ for 15 min at (**A**) pH 7.4 (*n* = 12/group, * *p* < 0.05) and (**B**) pH 5.9 (*n* = 7/group, * *p* < 0.05) before 2 μL of this solution was added to a 200 μL reaction mixture on a 96-well plate containing 250 μM ascorbic acid and 200 μM H_2_O_2_. (**C**) metMb was reacted with H_2_O_2_ at pH 5.9 as described for (**B**), and peroxidase activity was measured using 500 μM TMB and 200 μM H_2_O_2_ (*n* = 10/group). (**D**) MetMb (222 μM) was pre-reacted with 100 μM H_2_O_2_ for 30 min before 1 μL of this solution was added to a 200 μL reaction mixture containing 250 μM ascorbic acid and 200 μM H_2_O_2_ at pH 8.5. * *p* ≤ 0.05, *n* = 12/group.

Like many other heme proteins [31], horse metMb has been shown to form heme-to-protein cross-links upon treatment with hydrogen peroxide [10,32]. To assess the effects of heme-protein cross-links on metMb peroxidase activity, we measured the activity of H_2_O_2_-reacted metMb with 3,3′,5,5′-tetramethylbenzidine (TMB) as well as ascorbic acid. Interestingly, pre-treatment of metMb with H_2_O_2_ at pH 7.4 significantly increased its peroxidase activity with ascorbic acid (Figure 1A). Further, this effect was more pronounced when the pre-treatment was performed at a pH of 5.9 (Figure 1B). When TMB was used as a substrate, however, pre-treatment had no effect on Mb peroxidase activity (Figure 1C). Given previous reports that heme-protein cross-links in H_2_O_2_-treated metMb are more readily formed at a lower pH [7], these data suggested to us that reaction of Mb with H_2_O_2_ might result in the formation of modified species with unique peroxidase activities. To further test this hypothesis, we sought to measure the peroxidase activity of H_2_O_2_-reacted metMb in an alkaline pH (pH 8.5) in which metMb has no peroxidase activity with ascorbate as substrate (Figure 1D). Interestingly, H_2_O_2_-reacted metMb retained peroxidase activity even under these alkaline conditions (Figure 1D), suggesting that H_2_O_2_-reacted metMb possesses distinct peroxidase activities relative to metMb.

**Figure 2:**
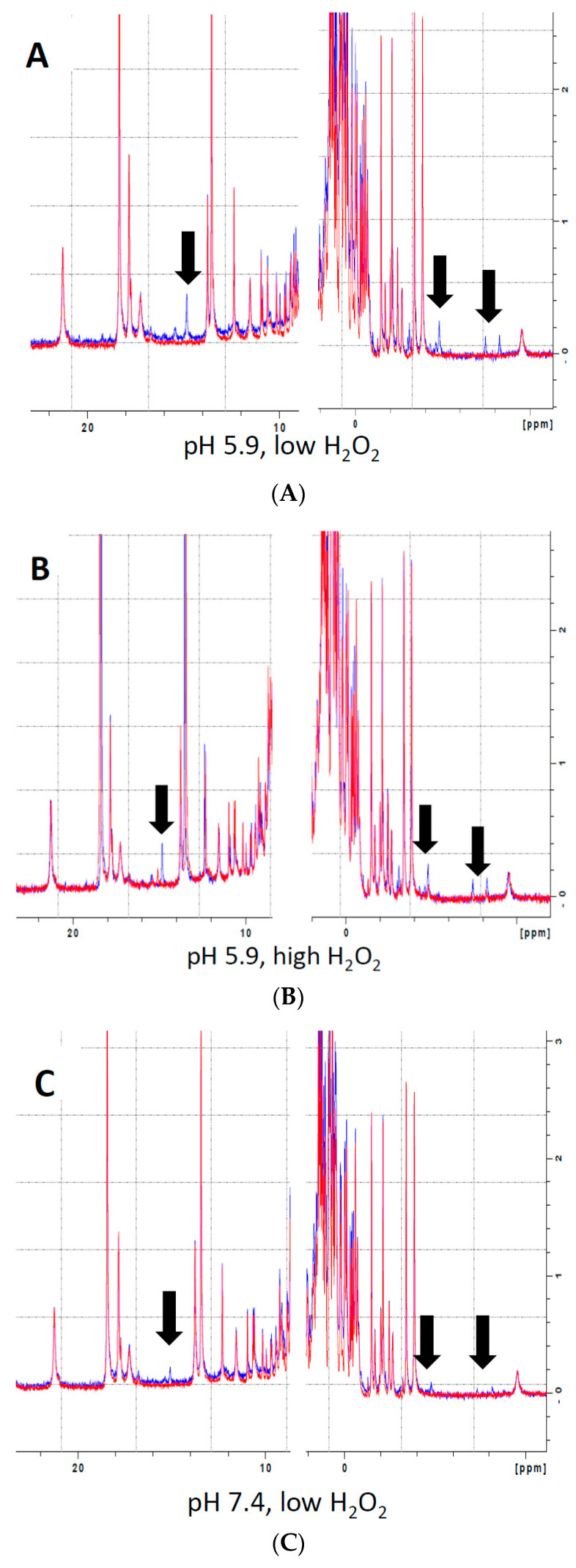
Analysis of heme-protein crosslinks by 700 MHz ^1^H NMR spectra. (**A**–**B**) MetMb (555 µM, pH 5.9) was reacted with 110 µM H_2_O_2_ (**A**) or 1.1 mM H_2_O_2_ (**B**) for 10 min before adding 3 mM NaCN to generate low-spin Mb. Sample preparation and ^1^H NMR procedures are described in the methods section. (**C**) Same as (**A**) and (**B**), only metMb was reacted with 110 µM H_2_O_2_ at pH 7.4. Data for untreated metMb are in red, and data for metMb pretreated with H_2_O_2_ are in blue. Arrows indicate novel peaks in the heme region for H_2_O_2_-treated metMb.

Analysis of the effects of H_2_O_2_ on heme electronic structure in Mb via ^1^H NMR spectroscopy revealed the presence of multiple novel peaks in the heme region (Figure 2, black arrows), indicating the presence of covalent modifications to the heme itself or to residues in its immediate vicinity. The presence of several novel peaks in the heme region might indicate either the presence of individual Mb-X molecules with multiple modifications, or, alternatively, that reaction of metMb with H_2_O_2_ results in various distinct Mb-X species. Since pre-treatment at lower pH further enhanced the ascorbate peroxidase activity of Mb (Figure 1B,C), we hypothesized that increased concentration of Mb-X species might be responsible for these effects. Consistent with our hypothesis, reaction of metMb with H_2_O_2_ at pH 5.9 produced ed the same novel heme resonances as found in metMb that was reacted with H_2_O_2_ at pH 7.4, though the intensity of these novel peaks was substantially increased at pH 5.9 (Figure 2A–C). We then sought to determine whether reaction at a higher H_2_O_2_ concentration would result in increased Mb-X formation. To our surprise, the higher H_2_O_2_ concentration had no effect on the amount of Mb-X present, as indicated by identical 1D ^1^H NMR spectra for low and high H_2_O_2_ concentrations (Figure 2A,B).

### 3.2. Heme Activity Stains of H_2_O_2_-Reacted metMb

**Figure 3:**
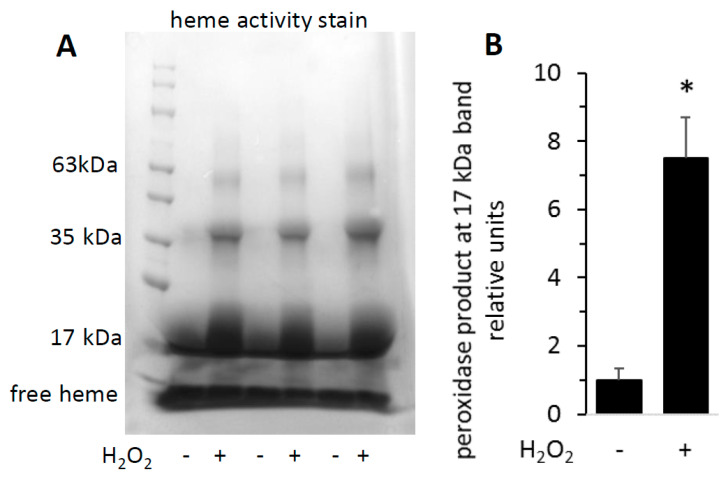
Reaction of metMb with H_2_O_2_ increases peroxidase activity as visualized on polyacrylamide gels. metMb (555 μM, pH 5.9) was incubated in the absence or presence of 800 μM H_2_O_2_ for 15 min before performing SDS-PAGE and (**A**) heme-peroxidase activity stains (right) as described in methods. (**B**) Quantitation of the peroxidase product at the 17 kDa band, *n* = 3/group, * *p* < 0.01.

To visualize the increased activity of H_2_O_2_-reacted metMb on a polyacrylamide gel, we subjected both untreated and H_2_O_2_-treated metMb to SDS-PAGE and stained the gels for heme peroxidase activity. As expected, the heme-stain bands were much more intense for H_2_O_2_-reacted Mb than the untreated control (Figure 3). Interestingly, exposure of Mb to H_2_O_2_ caused appearance of bands corresponding to the molecular weights of Mb dimers and trimers, an effect that will be addressed later in this study.

### 3.3. Unique Activity of Heme-Coupled Mb (Mb-X)

**Figure 4:**
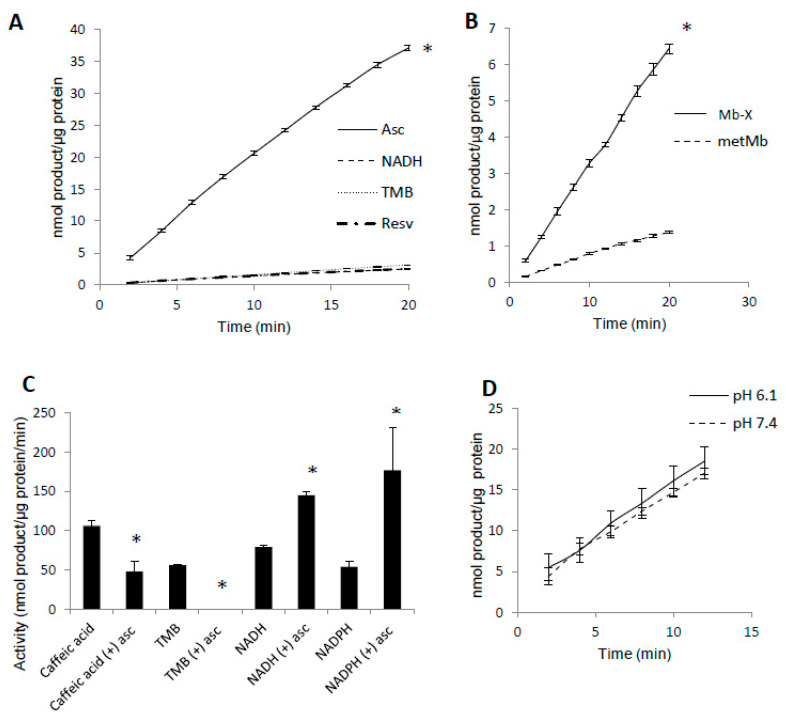
Mb-X species possess unique peroxidase activities compared to metMb. (**A**) Mb-X peroxidase activity was measured using ascorbic acid (Asc, 250 μM), resveratrol (Resv, 125 μM), NADH (250 μM) and TMB (500 μM) and 200 μM H_2_O_2_ at pH 6.1. The activity plots for all substrates except ascorbic acid overlap, so they are not all visible. *n* = 6/group, * *p* <0.05 vs. all other groups. (**B**) Mb-X concentration was estimated as described in the methods section. Peroxidase activity with ascorbic acid was then measured with 250 μM ascorbic acid and 200 μM H_2_O_2_ at pH 6.1. * *p* ≤ 0.05 compared to metMb, *n* = 3/group. (**C**) Mb-X peroxidase activity using caffeic acid (125 μM), TMB (500 μM) and NAD(P)H (250 μM) and 200 μM H_2_O_2_, pH 6.1 both in the presence and absence of 50 μM ascorbic acid. * *p* ≤ 0.05 compared to control without ascorbic acid, *n* = 3/group. (**D**) Mb-X peroxidase activity was measured at pH 7.4 and 6.1 using 250 μM ascorbic acid and 200 μM H_2_O_2_ (*n* = 3/group).

Since prior reaction with H_2_O_2_ increased metMb peroxidase activity, we hypothesized that most metMb activity is due to Mb-X species. To test this, we first treated metMb in conditions that favor Mb-X formation (as described in Section 2) and then removed non-covalently bound heme molecules with acid-butanone treatment. Next, we measured the activity of the Mb-X using several different substrates. Interestingly, Mb-X displayed unique peroxidase activities relative to metMb. Whereas metMb is a promiscuous peroxidase in terms of substrate selectivity [25], Mb-X displayed little to no activity with substrates other than ascorbic acid (Figure 4A). To quantitatively compare the activity of Mb-X to that of metMb, we estimated the concentration of heme-containing Mb molecules by measuring the amount of heme remaining in the organic phase after acid-butanone extraction. Using this estimated Mb-X concentration, we found Mb-X to possess four-fold greater activity with ascorbic acid compared to an equivalent concentration of metMb (Figure 4B).

In addition to switching Mb’s substrate preference for ascorbate, we found that pre-treating Mb-X with low concentrations (50 μM) of ascorbic acid actually enhanced its activity with NADH and NADPH, whereas its activity with other substrates (i.e., TMB and caffeic acid) was either strongly reduced or completely abolished by the same pre-treatment [25] (Figure 4C). We also found no difference in Mb-X peroxidase activity at pH 7.4 versus 6.1 (Figure 4D), although we previously showed that metMb peroxidase activity was increased nearly three-fold at pH 6.1 relative to 7.4 [25], suggesting that the pH dependence of metMb peroxidase activity might be explained by formation of Mb-X. 

### 3.4. Reversibility of Mb-X Species

**Figure 5:**
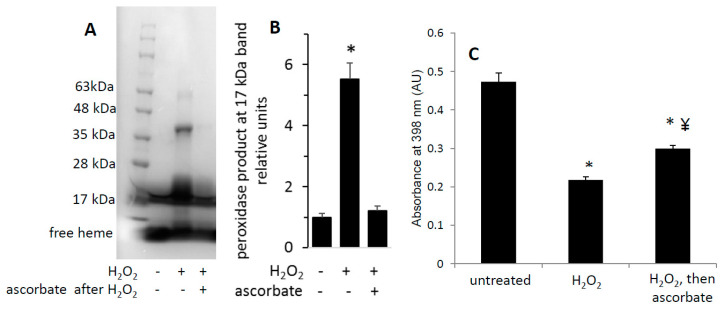
Heme-dependent reaction of H_2_O_2_-oxidized metMb with ascorbic acid is sufficient to reverse heme-protein crosslinks. (**A**) MetMb (111 μM, pH 6.1) was incubated in the absence or presence of 300 μM H_2_O_2_ for 10 min prior to adding 833 μM ascorbic acid for an additional 10 min and performing SDS-PAGE followed by (**A**) a heme peroxidase stain. (**B**) Quantitation of the peroxidase product at the 17 kDa band, *n* = 5/group, * *p* < 0.001. (**C**) Acid-butanone heme extraction was performed on metMb that was either untreated, reacted with H_2_O_2_ alone for 10 min (H_2_O_2_) or treated with ascorbic acid after 10 min of H_2_O_2_ oxidation. Free heme was assessed by absorbance at 398 nm. * *p* ≤ 0.05 compared to untreated control; ^¥^ *p* ≤ 0.05 compared to H_2_O_2_-only; *n* = 3/group.

Interestingly, H_2_O_2_-treated metMb samples that were incubated with ascorbic acid prior to SDS-PAGE did not retain an increased heme activity stain (Figure 5A,B), suggesting that, in the presence of excess ascorbic acid, Mb could reverse its cross-linkage to heme. To test this hypothesis, we treated H_2_O_2_-reacted metMb with ascorbic acid and then measured the corresponding amount of heme that was lost after acid-butanone treatment. In support of our hypothesis, treating H_2_O_2_-reacted metMb with ascorbic acid prior to acid-butanone treatment significantly increased the corresponding amount of free heme (Figure 5C), indicating that ascorbic acid partially reversed the heme:protein cross-link under these conditions.

**Figure 6:**
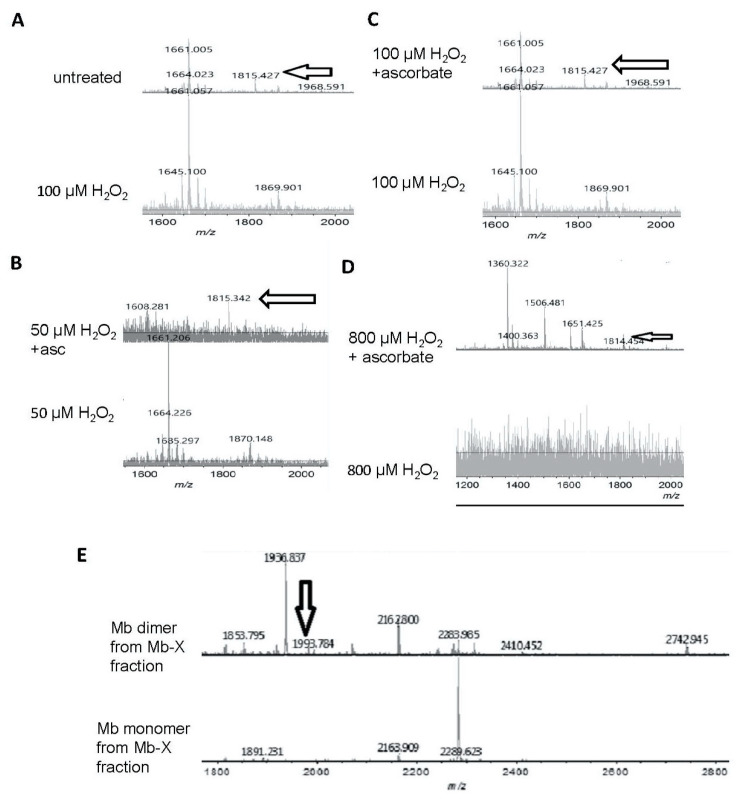
Matrix assisted laser desorption mass spectrometry-time of flight (MALDI-TOF) mass spectrometric analysis of ascorbic acid-mediated reversal of heme-protein crosslinks in H_2_O_2_-reacted metMb. (**A**) (Bottom spectra) MetMb tryptic digests that were reacted with 100 μM H_2_O_2_ displayed loss of the N-terminal peptide (1815.9 *m*/*z*) indicated by arrows in the untreated MetMb (top spectra). (**B**–**D**) Tryptic digests of H_2_O_2_-reacted metMb that was then treated with ascorbic acid revealed the re-appearance of the N-terminal peptide. (**E**). MetMb monomer (bottom) and dimer (top) tryptic digests that were reacted with 300 µM H_2_O_2_ and analyzed with MALDI-TOF MS. The arrow indicates a peak at 1993 *m*/*z*, which suggests presence of a heme crosslinked to a peptide containing the distal histidine of Mb helix E.

To investigate the reversibility of H_2_O_2_-induced modifications of Mb, we analyzed H_2_O_2_-treated metMb with and without ascorbic acid treatment using matrix assisted laser desorption mass spectrometry-time of flight (MALDI-TOF) mass spectrometry. Tryptic digests of H_2_O_2_-reacted metMb showed a missing peak at 1815 *m*/*z* (Figure 6A), suggesting that this peptide could participate in a cross-link. The peptide at 1815 *m*/*z* re-appeared in the ascorbic acid-treated tryptic digests of Mb that had been initially incubated with various H_2_O_2_ concentrations (50 μM, 100 μM, and 800 µM (Figure 6B–D)), indicating that ascorbic acid-treatment was sufficient to reverse this cross-linked species. The tryptic peptide corresponding to the 1815 *m*/*z* peak contains a tryptophan (position in peptide denoted by underlining, GLSDGEWQQVLNV**W**GK), which is known to harbor a radical upon reaction with H_2_O_2_ [33], making it a plausible candidate for oxidative modification or cross-linking.

Remarkably, after subjecting metMb to harsh oxidation conditions (800 μM H_2_O_2_ for 30 min at pH 5.9), no tryptic peptides were detected above the signal-to-noise threshold (Figure 6D), indicating that these stringent conditions had severely modified metMb. After these samples were treated with excess ascorbic acid, however, multiple peptides re-appeared (Figure 6D).

In addition to forming Mb-X when treated with H_2_O_2_, Mb also forms dimers as is visible in Figure 3A. We extracted Mb-X from H_2_O_2_-treated Mb, separated the products SDS-PAGE, gels, and performed MALDI-TOF on tryptic digests of the Mb monomer and dimer. We found a novel peak at 1993.8 *m*/*z* present in the H_2_O_2_-reacted Mb dimer (Figure 6E) that amounts to theoretical mass of a heme-crosslinked Mb peptide when accounting for the loss of two protons from the crosslinking reaction (1378.8 + 616.5 − 2.0 Da). Notably, this peptide contains the distal histidine (position in peptide denoted by underlining, **H**GTVVLTALGGILK), which has previously been implicated in heme-protein crosslinks in H_2_O_2_-treated Mb [8].

### 3.5. Complex Substrate Specificity of metMb Peroxidase Activity

**Figure 7:**
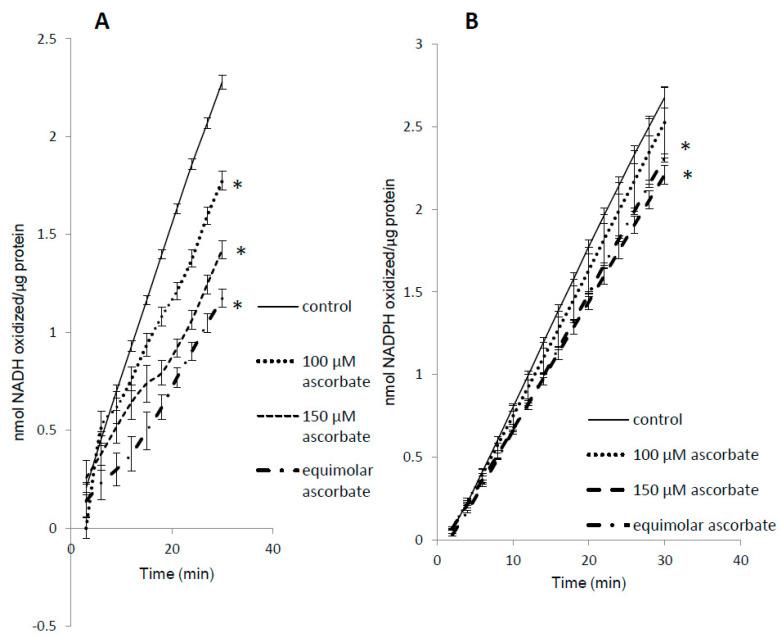
Substrate competition for metMb peroxidase activity. MetMb peroxidase activity with NADH (**A**) or NADPH (**B**) was measured in the presence of varying concentrations of ascorbic acid. * *p* ≤ 0.05 compared to NAD(P)H without ascorbic acid present. *n* = 6/group in (**A**) and *n* = 11–12/group in (**B**).

We found that ascorbic acid differentially competed with NADH and NADPH as substrates for metMb (Figure 7A,B). Even at equimolar concentrations of ascorbic acid, the rate of NADPH oxidation was minimally affected (Figure 7B), whereas the same ratio of ascorbic acid reduced metMb peroxidase activity with NADH by ~50% (Figure 7A). Notably, we have previously shown that metMb has similar peroxidase activity [25] with NADH and NADPH, which would imply that they both should be equally affected by the same competing substrate.

### 3.6. H_2_O_2_-Dependent Dimerization of metMb

**Figure 8:**
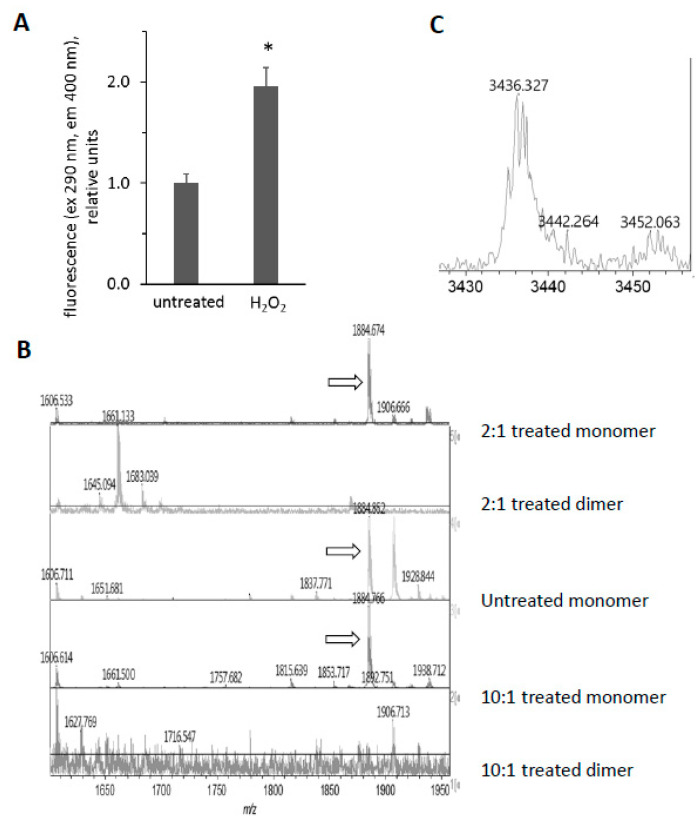
Reaction of horse metMb with H_2_O_2_ results in dityrosine formation. (**A**) Fluorescence of metMb was measured before and after oxidation with H_2_O_2_ using excitation and emission wavelengths of 290 nm and 400 nm, respectively. * *p* ≤ 0.005 compared to untreated, *n* = 6/group. (**B**) MALDI-TOF analysis of metMb tryptic digests revealed that the tryptic peptide containing tyrosine 103 (1885 *m*/*z*) was absent in the H_2_O_2_-treated dimer. From bottom: dimer from 222 μM Mb reacted with 2.2 mM H_2_O_2_; monomer from 222 μM Mb reacted with 2.2 mM H_2_O_2_; untreated monomer; 222 μM Mb dimer reacted with 444 μM H_2_O_2_; 222 μM Mb monomer reacted with 444 μM H_2_O_2_. Arrows indicate peaks for 1885 *m*/*z*. (**C**) MALDI-TOF analysis of oxidized metMb dimers indicate the presence of a Y103-Y146 dityrosine cross-linked peptide with a *m*/*z* of 3436.3.

We were intrigued by the appearance of high molecular weight bands after treatment of Mb with H_2_O_2_ and the disappearance of these bands after subsequent exposure to ascorbic acid (Figure 3 and Figure 5). Since horse Mb does not contain cysteine residues and thus the crosslinks could not be disulfide bonds, we sought to determine the nature of the oxidative cross-link. Previous reports [34] have found dityrosine cross-links in the H_2_O_2_-treated Mb dimer. Since dityrosine has a unique fluorescence spectrum, we first measured the fluorescence of H_2_O_2_-treated metMb (Figure 8A). Consistent with the presence of dityrosine, H_2_O_2_-reacted Mb samples displayed significantly increased fluorescence corresponding to the excitation and emission wavelengths in the range of the dityrosine spectra [17]. Mass spectrometric analysis showed that the peptide containing tyrosine 103 is absent in the H_2_O_2_-treated dimer, while it is present in both the treated and untreated monomer (1885 *m*/*z*, Figure 8B). This corresponds to the tryptic peptide (K)YLEFISDAIIHVLHSK(H) containing Y103. In addition, a novel peptide at 3436.4 *m*/*z* appeared in the H_2_O_2_-reacted dimer (Figure 8C). Presumably, this is a species with the peptide containing Y103 cross-linked to the C-terminal tryptic missed cleavages peptide containing Y146, (R)NDIAAKYKELGFQG(-). The 3436.4 *m*/*z* peak corresponds to the theoretical mass of a Y103-Y146 cross-link (1885.0 *m*/*z* + 1553.8 *m*/*z* − 2 protons lost). Although these results do not rule out the possibility of other cross-links, they strongly indicate the presence of dityrosine cross-links in H_2_O_2_-reacted horse Mb. 

### 3.7. Reversal of Protein-to-Protein Cross-Links

**Figure 9:**
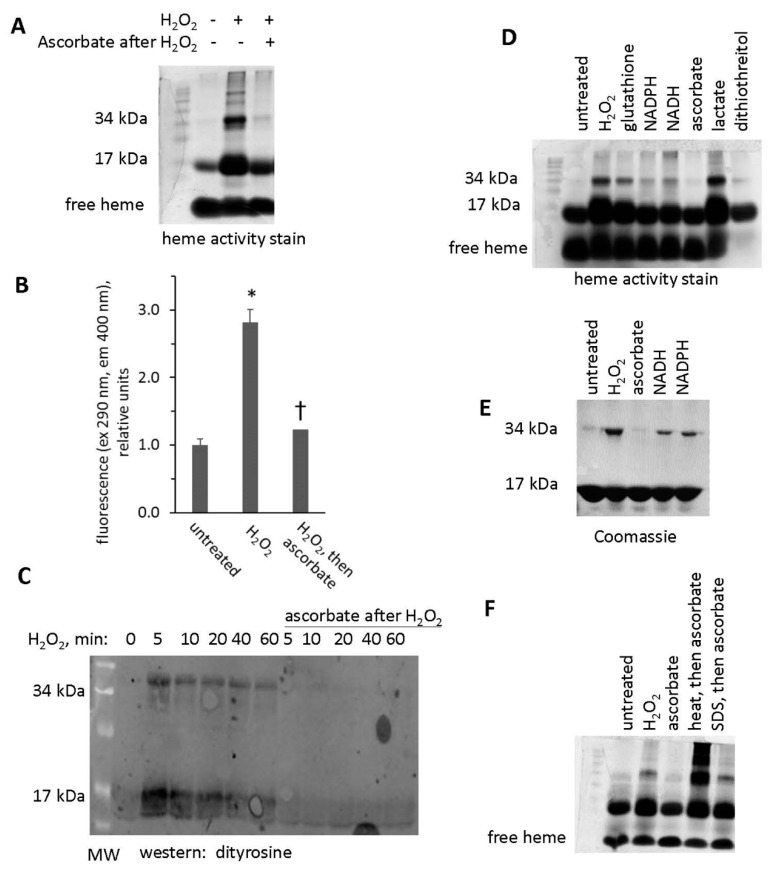
Treatment with ascorbic acid reverses crosslinking of Mb dimers. (**A**) Heme peroxidase activity stain of metMb that was untreated, H_2_O_2_-treated, or H_2_O_2_-treated followed by exposure to ascorbic acid. (**B**) Fluorescence of metMb treated as for panel A was assessed at excitation 290 nm and emission 400 nm, which correspond to the fluorescence characteristics of dityrosine [17], *n* = 8/group, * *p* < 0.001 vs untreated and group treated with peroxide and then ascorbic acid, † *p* = 0.05 vs untreated. (C) Western blot of ascorbic acid-treated oxidized metMb using an anti-dityrosine antibody. (D) Heme stain of H_2_O_2_-reacted metMb that was then treated with various reducing substrates. (E) Coomassie stain of Mb exposed to H_2_O_2_ and then reducing agents. (F) Heme stain of oxidized metMb that was denatured by heating for 10 min at 90 °C or by adding SDS-PAGE sample loading buffer prior to the addition of ascorbic acid.

Data discussed above suggests that H_2_O_2_ can induce protein-to-protein cross links in Mb and that subsequent treatment with ascorbic acid can break the crosslinks. This is shown for samples subjected to a heme peroxidase stain after exposure of Mb to H_2_O_2_ and then ascorbic acid (Figure 9A). For data shown in Figure 9A, we let the peroxidase stain proceed long enough to develop multiple bands at high molecular weights, corresponding to Mb dimers, trimers, and larger aggregates. We found that ascorbic acid treatment significantly reduced the fluorescence of H_2_O_2_-reacted metMb (Figure 9B), which is consistent with the idea that ascorbic acid facilitates the cleavage of Mb’s protein-to-protein crosslinks. 

Western blot analysis using an anti-dityrosine antibody suggests that intra- and intermolecular dityrosine is present in H_2_O_2_-reacted metMb monomer and dimer, respectively (Figure 9C). Treatment of H_2_O_2_-reacted metMb with ascorbic acid eliminated reactivity with the antibody against dityrosine. In addition, treatment with other biological reducing substrates (glutathione, NADPH, NADH, and dithiothreitol) also reversed the dimer, although to differing extents (Figure 9D,E). 

We next sought to determine the extent to which the Mb protein itself was responsible for cleavage of protein-to-protein bonds. To do this, we subjected oxidized metMb to both heat and detergent denaturation prior to ascorbic acid treatment. As shown in Figure 9F, heat and detergent-mediated denaturation inhibited the ability of ascorbic acid treatment to reverse metMb dimers, thus confirming that the native Mb protein plays a role in the mechanism of breaking protein-to-protein crosslinks.

### 3.8. Potential Role of Tyrosine Residues in Breaking Mb–Mb Cross-Links

**Figure 10:**
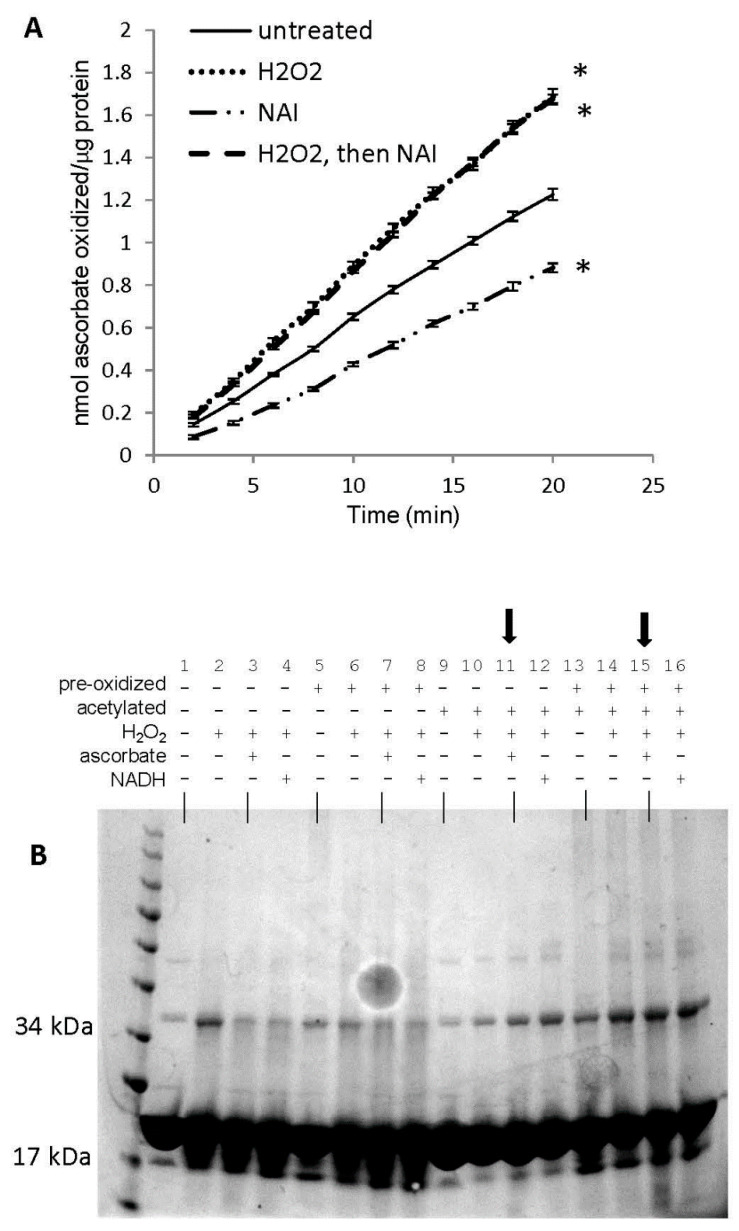
Role of tyrosine residues in regulating oxidatively-modified metMb species. (**A**) Peroxidase activity assay of metMb that had been treated with H_2_O_2_ alone, N-acetylimidazole (NAI) alone or H_2_O_2_ and then NAI (as described in methods and materials) using 200 μM H_2_O_2_ and 250 μM ascorbic acid at pH 6.1. * *p* ≤ 0.05 compared to untreated control, *n* = 6/group. Activity plots for H_2_O_2_ and NAI groups overlap, so they are not both visible. (**B**) Coomassie stains of the samples prepared in (**A**). Lanes 1–4: untreated, control metMb; H_2_O_2_-treated, control metMb; H_2_O_2_-treated control metMb + ascorbic acid; H_2_O_2_-treated control metMb + NADH. Lanes 5–8: same as (1–4) only using met Mb that had been reacted with H_2_O_2_ (pre-oxidized). Lanes 9–12: same as (1–4) and (5–8) only using NAI-treated metMb. Lanes 13–16: same only using H_2_O_2_-treated then NAI-treated metMb. Lanes showing that tyrosine-acetylated Mb cannot reverse Mb dimerization in the presence of ascorbic acid are indicated by arrows.

We have previously shown that acetylation of tyrosine residues in horse metMb differentially affects its peroxidase activity depending on the reducing co-substrate used [25]. We hypothesized that this discrepancy might be due to tyrosine-mediated formation of oxidatively-modified Mb species (i.e., metMb monomer, metMb dimer, Mb-X monomer, Mb-X dimer, etc.) with different peroxidase activities with different reducing co-substrates. As displayed in Figure 10A, tyrosine acetylation significantly decreased the peroxidase activity of metMb using ascorbic acid as a reducing co-substrate. However, the inhibitory effect of tyrosine acetylation was completely abrogated if metMb was first pre-treated with H_2_O_2_, suggesting that the role of the tyrosine residues is directing crosslinks as opposed to directly participating in redox cycling. In addition, the activity of metMb that was first treated with H_2_O_2_ and then acetylated was nearly identical to the activity of metMb that was only pre-treated with H_2_O_2_, indicating that the increased activity-conferring oxidative modifications had already occurred prior to acetylation.

SDS-PAGE of these samples revealed that both acetylated (with or without pre-H_2_O_2_ treatment) and unacetylated metMb could form dimers upon reaction with H_2_O_2_ (Figure 10B). However, neither acetylated sample could reverse its dimer(s) after treatment with ascorbic acid, indicating that tyrosine residues are not necessary to form dimers but that they are necessary for reversing them. 

## 4. Discussion

The new information provided by this study includes the novel findings of reversible oxidative modifications of Mb upon treatment with ascorbic acid. For example, exposure to H_2_O_2_ increases Mb peroxidase activity and preference for ascorbate as the reducing co-substrate for Mb peroxidase activity. This increase of peroxidase activity was associated with Mb-X formed by reaction of Mb with H_2_O_2_, and both the increase in peroxidase activity and the Mb-X crosslink were reversed by treatment with ascorbic acid. While H_2_O_2_-reacted Mb forms intramolecular crosslinks to form dimers, trimers, and larger Mb aggregates, an important novel finding of the current study is that these interprotein bonds are broken by treatment with ascorbate. This action does not occur if the aggregates are first denatured by heat or incubation with SDS, suggesting that the native protein plays a role in reversal of interprotein crosslinks.

While the increase in peroxidase activity caused by exposure of Mb to H_2_O_2_ is a novel finding, there are reports of other Mb redox activities being enhanced by reaction of Mb with H_2_O_2_. For example, treatment of sperm whale or horse Mb with H_2_O_2_ reportedly increases NADH oxidase activity by up to 20-fold, and this activity when assessed for horse Mb was associated with Mb-X [12]. The stoichiometry of this reaction was 1 mol of NADH oxidized per 1 mol of O_2_ consumed, which is consistent with a two-electron transfer from NADH to O_2_, forming H_2_O_2_ [12]. Osawa and Korzekwa suggested that this NADH oxidase activity of Mb-X could contribute to toxicity by promoting further production of H_2_O_2_ [12]. Other deleterious reactions mediated by H_2_O_2_-reacted Mb or Mb-X include peroxidation of lipids, phospholipids, LDL, and cholesterol esters [13,35]. Holt et al. reported increased presence of Mb-X and the free-radical-induced peroxidation of arachidonic acid, F2-isoprostanes, in urine of patients with rhabomyolysis [16], suggesting a central role of Mb-X in rhabodomyolysis-related tissue damage. In contrast to the reactions described above, which are oxidative in nature (i.e., either promoting peroxidation or producing H_2_O_2_), an increase in peroxidase activity as shown in the current study would be a means to counteract an increase in reactive oxygen species.

Although myoglobin in the presence of H_2_O_2_ can produce hydroxyl radical, scavengers of hydroxyl radical had no effect on peroxidation of either uric acid or arachidonic acid peroxidation in the presence of Mb and H_2_O_2_ [35]. This suggests that the Mb itself—as opposed to Mb-produced hydroxyl radical—mediates uric acid or acachidonic peroxidation. In contrast, sulfhydryl reducing agents can prevent peroxidation of uric acid or arachidonic acid by myoglobin in the presence of H_2_O_2_ [35]. A suggested mechanism of the protective effects of the reducing agents was that they prevented formation of a reactive derivative of Mb [35], such as Mb-X. Mb-X, originally known as the green-pigmented species formed by reaction of Mb with H_2_O_2_, was found to be stable in solution at room temperature for months [11]. The new information provided by the current study is that reducing agents can reverse—as opposed to simply prevent—formation of Mb-X. Because it seems apparent that Mb-X plays a toxic role in conditions such as rhabdomyolytis [15,16], this novel demonstration of reversal of the Mb-X crosslink by ascorbic acid has important therapeutic implications.

Catalano et al. reported that treatment of horse Mb with H_2_O_2_ causes a ~50% decrease in tyrosine (Y) content [10]. Tryptic digests of H_2_O_2_-treated Mb contained a species not present in untreated Mb that had a molecular weight consistent with a heme group covalently bound to a peptide beginning at Y103 (YLEFISDAIIHVLHSK), though this peptide had virtually no Y content in H_2_O_2_-treated Mb compared to untreated Mb [10]. Taking these data together, the authors suggested that reaction with H_2_O_2_ produces Mb with the heme covalently bound to Y103 [10]. Reeder et al. [8] re-examined the hypothesis that Y103 mediates the heme-to-Mb cross link by using site-directed mutagenesis of sperm whale Mb. While the Y103F mutation did not affect Mb-X formation, the H64V mutation of the E7 helix distal histidine almost fully prevented generation of Mb-X [8]. Consistent with this, wild-type *Aplysia limacina* that lacks the E helix distal histidine does not form Mb-X, while introduction of a histidine residue into *aplysia* promotes Mb-X formation [8]. We have detected a tryptic fragment of H_2_O_2_-reacted Mb that is consistent with the analysis of Reeder et al. [8] that the cross-link of Mb-X can be mediated by histidine. 

Reeder et al. reported that H_2_O_2_ reaction with Mb forms Mb-X at increasing rates when pH decreases [36]. The authors suggested that formation of Mb-X requires both the protonated oxoferryl heme and a protein radical. It also appears that a protein radical is required for protein-to-protein Mb cross linking [37]. Detweiler et al. used 3,4-dihydro-2,3-dimethyl-2H-pyrrole 1-oxide (DMPO) to trap radicals formed by reaction of sperm whale Mb with H_2_O_2_ [37]. DMPO prevented formation of sperm whale Mb dimers and trimers [37]. Use of DMPO followed by electrospray mass spectrometry showed that a peptide containing Y103 was the site of the radical [37]. This is consistent with the suggestion of Svistunenko et al. that given the proximity of Y103 to the heme, the radical originates on Y103 before passing to other sites in Mb [38]. Iodinization of horse Mb prevented subsequent formation of Mb dimers by reaction with H_2_O_2_, consistent of a role of a role of tyrosinyl side chains in generation of protein-to-protein cross links [37]. Further, Y151 of sperm whale Mb, a tyrosine lacking in horse Mb, was required for Mb dimer formation [37]. Our findings stand in contrast to those of Detweiler et al. [37] in that tyrosine acetylation did not affect Mb dimer formation in the current study, suggesting that tyrosine-independent crosslinks can contribute to the Mb aggregation in the current study. 

Mb-X formation from metMb is modestly faster than Mb-X formation from oxygen-bound Mb (oxyMb) [36]. However, Mb-X formation from metMb is inhibited by presence of oxyMb [36]. This brings into question whether Mb-X formation could occur intracellularly under normoxic conditions in which oxyMb would be the predominant Mb form. On the other hand, it has been shown by magnetic resonance spectroscopy of human skeletal muscle that moderate aerobic exercise (about 50% or 60% of maximum oxygen consumption rate ($\dot{V}$O_2_max) causes about 50% of Mb to be in its deoxygenated form (deoxyMb) in the contracting skeletal muscle [39,40]. This deoxygenation of Mb sets in at moderate exercise intensity but does not further increase as exercise intensity increases up to $\dot{V}$O_2_max [39,40]. At the same time that exercise increases deoxyMb [39,40], muscle contractions also increase intracellular NADPH oxidase-generated superoxide [41], which rapidly dismutates to H_2_O_2_. Thus, it appears that aerobic exercise could create conditions under which Mb-X could potentially form (i.e., increases in both H_2_O_2_ and deoxyMb).

The dityrosine western blot showing reversal of dityrosine cross-links should be treated with caution, as dityrosine has such a high bond dissociation energy [42] that it would be unlikely to be broken. Consistent with a side chain other than tyrosine participating in protein crosslinking, acetylation of tyrosine residues did not prevent formation of Mb dimers. As a potential alternative mechanism of Mb–Mb cross-linking, it is possible that some other amino acid side chain, such as tryptophan, participates in protein-to-protein linkages. For example, radical-induced generation of ditryptophan (W–W) and tryptophan-tyrosine (W–Y) crosslinks has been shown to mediate protein and peptide dimerization [43,44,45]. W–W crosslinks have a fluorescence excitation and emission profile [46] that is similar to that of dityrosine [17] and so could potentially contribute to the fluorescence changes (e.g., Figure 8A and Figure 9B) in Mb exposed to H_2_O_2_. Mb contains a tryptophan residue that becomes a tryptophanyl radical after reaction with H_2_O_2_ [33]. Our MALDI-TOF data show that the peptide containing this W residue (and also the other W in horse Mb) disappears after treatment of Mb with H_2_O_2_ and reappears after subsequent treatment with ascorbic acid. Given the position of this W residue in Mb, it seems unlikely to be able to participate in binding to heme. However, the data are consistent with W being a candidate for forming reversible protein-to-protein crosslinks between Mb proteins, perhaps involving W–W bonds, W–Y bonds, or both. Our findings suggest the vital importance of denaturing H_2_O_2_-reacted Mb with heat and/or SDS before running blots under reducing conditions to ensure both detection of Mb dimers and larger aggregates and subsequent reversal, given the labile nature of the cross links when exposed to reducing agents in the presence of native (i.e., non-denatured) Mb.

The nature of ditryptophan bonds is not yet fully elucidated [47]. Available data suggest that C and N participate in ditryptophan crosslinking [47], giving the possibility of either C–C or C–N crosslinks. Notably, the ditryptophan dimer in superoxide dismutase or lysozyme can cleave under MS/MS conditions [47], suggesting that it is not as stable as a dityrosine link and thus is similar to the labile crosslinks we have detected in the current study. Paviani et al. have suggested that the susceptibility to cleavage of ditryptophan is more consistent with a C–N bond than a C–C bond [47]. Accordingly, the crosslink that is reversed by ascorbate in the current study is likely a C–N bond.

Our finding of a peptide corresponding to the mass of a peptide containing both Y103 and Y146 suggests that both tyrosines can participate in dityrosine crosslinks. We did not collect data on which tyrosine residues were acetylated by treatment of Mb with N-acetylimidazole. When horse heart Mb is incubated with a 100-fold excess of N-acetylimidazole, both tyrosines are acetylated, though the amount of tyrosine acetylated can be varied by titration with N-acetylimidazole and assessed by changes in tyrosine absorbance at 280 nm [48]. Our N-acetylimidazole-to-Mb molar ratio was 10-fold lower than that used by Giulivi et al. [49], so it is possible that Y103 was unaffected under these conditions. The finding that dimerization was not prevented by acetylation suggest that Y103 was not acetylated by N-acetylimidazole in our study. For example, Y103 is likely the site of radical initiation before the radical is passed to other residues [38], which would be necessary for radical-induced dimerization involving residues such as tyrosine or tryptophan. Interestingly, iodination of horse heart Mb prevents Mb dimerization [37]. This suggests that the dimerization reported by Detweiler et al. [37] is truly mediated by dityrosine, as opposed to the labile crosslink found in the current study.

Y103 is local to the heme, and it appears that formation of a radical occurs at Y103 before transferring to other residues [37,38]. We have detected a peptide containing both Y103 and Y146 in H_2_O_2_-treated Mb. This suggests that both the heme-localized tyrosine and the tyrosine in a helix closer to the protein surface can participate in dityrosine bonding. The distal histidine is in close proximity to the heme and has previously been demonstrated to form a cross link with heme [8]. W14, on the other hand, is distant from both the heme and Y103 but still reportedly forms a radical when Mb reacts with H_2_O_2_ [33]. For reference regarding positions of the heme and amino acids in Mb, a 3D structure of horse heart metMb (MMDB ID 57734) is available in the Molecular Modeling Database (MMDB) [50] housed by the National Center for Biotechnology Information.

Dityrosine occurs in various functional, structural elements such as silk proteins [51,52], elastin [53], and sea urchin eggs [54]. On the other hand, dityrosine can be a marker for both aging-associated oxidative damage [55] and acute bouts of oxidative stress, such as in myocardial infarction [56]. While the aforementioned studies consider dityrosine to be a biomarker for oxidative stress, others have proposed that it might play more of a harmful role in certain mammalian tissues. For example, dityrosine-mediated cross-linking of β-amyloid peptide [23,57] and α-synuclein protein [24] promotes the stabilization of their respective aggregates. Interestingly, overexpression of neuroglobin (Ngb), an oxygen-binding globin expressed mainly in neurons, has been shown to reduce Aβ fibril formation in vivo [58], and low Ngb levels correlate with Alzheimer’s disease [59]. The data from the current study suggest that ascorbic acid can break protein-to-protein crosslinks caused by reaction of Mb with H_2_O_2_. Thus, it seems possible that actions of globins might be a means through which deleterious protein aggregates could be broken in vivo. 

Reaction schemes for reduction of ferrylMb (Mb with Fe^4+^ in an oxo complex) by ascorbate such as would occur in peroxidase activity [60], pH dependence of Mb redox reactions [61], generation of Mb-X [36], formation of dityrosine [17], and formation of ditryptophan [43,47] are presented in the literature. The increased peroxidase activity once Mb is treated with H_2_O_2_ is most likely due to formation of the heme-to-protein crosslink. The mechanism for the increased redox activity of Mb-X has not been elucidated, but it has been suggested to be attributable to a change in protein structure surrounding the heme [12]. Although we do not know the mechanism by which ascorbate becomes a preferred reducing substrate of Mb-X, we speculate that Mb-X retains the ability to be reduced by ascorbate at both sites for electron donation on Mb as described by Reeder et al. [60].

Translation of the in vitro data presented in this study to physiological conditions relies on the assumption that metMb would be present in vivo. While Mb protein is expressed in mammalian heart and skeletal muscle at about 400 µmol/kg [62,63], metMb concentration is relatively low in tissues in vivo if it is present at all. For example, Kreutzer et al. reported that metmyoglobin is undetectable by NMR in perfused rat heart [64]. On the other hand, it has been suggested that 1% of Mb in cardiac tissue is in the metMb form [65]. As measured spectroscopically in anoxic pig heart, metMb content was 32 µmol/dm^3^, which was about 6% of total Mb [66]. Ascorbate concentrations in human skeletal muscle are about 170 µmol/kg [67]. Unfortunately, H_2_O_2_ levels in skeletal muscle have been difficult to measure [68]. Palomero et al. [69] used extracellular H_2_O_2_ to calibrate intracellular H_2_O_2_ levels in isolated rat skeletal muscle fibers using the reactive oxygen species probe chloromethyl-2′,7′-dichlorofluorescin. They used this extracellular H_2_O_2_ standard to estimate that intracellular H_2_O_2_ reached about 1 µM during contractile activity [69]. Jackson later suggested that this H_2_O_2_ concentration would be closer to 0.1 µM due to a trans plasma membrane H_2_O_2_ gradient that would result from H_2_O_2_ being applied to the extracellular medium [68]. In summary, the in vitro concentrations of metMb and H_2_O_2_ used in the current study are much greater than would be found in the intracellular environment. Future study should be done to determine whether reversible modifications of Mb, such as Mb-X or labile Mb aggregates, can be found in skeletal muscle or heart under normal and pathological conditions. Of course, Mb can be also be found extracellularly, in conditions such as rhabdomyolysis. In the extracellular milieu, where serum H_2_O_2_ concentration and plasma ascorbate concentration are both about ~50 µM [67,70], the reversible modifications described in the in vitro studies of the current paper seem possible. Future work should investigate whether reversible modifications of Mb occur in vivo in both intracellular and extracellular spaces.

## 5. Conclusions

Collectively, our data indicate a redox role for Mb. We demonstrate that formation of heme-protein crosslinked species substantially increases Mb peroxidase activity as well as specificity with ascorbate as a reducing co-substrate. These findings point toward a potential regulatory role of ascorbate for Mb peroxidase activity in oxidatively-challenged muscle and heart. We also show that both heme-to-protein and protein-to-protein crosslinks in horse Mb can be broken by treatment with ascorbic acid, which indicates that Mb might play a role to reverse oxidative protein modifications.

## References

[1] Stadtman E.R., Levine R.L. (2003). Free radical-mediated oxidation of free amino acids and amino acid residues in proteins. Amino Acids.

[2] Boronat S., Garcia-Santamarina S., Hidalgo E. (2015). Gel-free proteomic methodologies to study reversible cysteine oxidation and irreversible protein carbonyl formation. Free Radic. Res..

[3] Reeg S., Grune T. (2015). Protein oxidation in aging: Does it play a role in aging progression?. Antioxid. Redox Signal..

[4] Kim N.H., Jeong M.S., Choi S.Y., Kang J.H. (2006). Oxidative modification of cytochrome c by hydrogen peroxide. Mol. Cells.

[5] Xiang W., Weisbach V., Sticht H., Seebahn A., Bussmann J., Zimmermann R., Becker C.M. (2013). Oxidative stress-induced posttranslational modifications of human hemoglobin in erythrocytes. Arch. Biochem. Biophys..

[6] Archakov A.I., Zgoda V.G., Karuzina I.I. (1998). Oxidative modification of cytochrome P450 and other macromolecules during its turnover. Vopr. Med. Khim..

[7] Giulivi C., Cadenas E. (1994). Ferrylmyoglobin: Formation and chemical reactivity toward electron-donating compounds. Methods Enzym..

[8] Reeder B.J., Cutruzzola F., Bigotti M.G., Watmough N.J., Wilson M.T. (2007). Histidine and not tyrosine is required for the peroxide-induced formation of haem to protein cross-linked myoglobin. IUBMB Life.

[9] Svistunenko D.A., Reeder B.J., Wankasi M.M., Silaghi-Dumitrescu R.L., Cooper C.E., Rinaldo S., Cutruzzola F., Wilson M.T. (2007). Reaction of Aplysia limacina metmyoglobin with hydrogen peroxide. Dalton Trans..

[10] Catalano C.E., Choe Y.S., Ortiz de Montellano P.R. (1989). Reactions of the protein radical in peroxide-treated myoglobin. Formation of a heme-protein cross-link. J. Biol. Chem..

[11] Fox J.B., Nicholas R.A., Ackerman S.A., Swift C.E. (1974). A multiple wavelength analysis of the reaction between hydrogen peroxide and metmyoglobin. Biochemistry.

[12] Osawa Y., Korzekwa K. (1991). Oxidative modification by low levels of HOOH can transform myoglobin to an oxidase. Proc. Natl. Acad. Sci. USA.

[13] Vuletich J.L., Osawa Y., Aviram M. (2000). Enhanced lipid oxidation by oxidatively modified myoglobin: Role of protein-bound heme. Biochem. Biophys. Res. Commun..

[14] Osawa Y., Williams M.S. (1996). Covalent crosslinking of the heme prosthetic group to myoglobin by H2O2: Toxicological implications. Free Radic. Biol. Med..

[15] Boutaud O., Moore K.P., Reeder B.J., Harry D., Howie A.J., Wang S., Carney C.K., Masterson T.S., Amin T., Wright D.W. (2010). Acetaminophen inhibits hemoprotein-catalyzed lipid peroxidation and attenuates rhabdomyolysis-induced renal failure. Proc. Natl. Acad. Sci. USA.

[16] Holt S., Reeder B., Wilson M., Harvey S., Morrow J.D., Roberts L.J., Moore K. (1999). Increased lipid peroxidation in patients with rhabdomyolysis. Lancet.

[17] Malencik D.A., Anderson S.R. (2003). Dityrosine as a product of oxidative stress and fluorescent probe. Amino Acids.

[18] Cheng G., Li H., Cao Z., Qiu X., McCormick S., Thannickal V.J., Nauseef W.M. (2011). Vascular peroxidase-1 is rapidly secreted, circulates in plasma, and supports dityrosine cross-linking reactions. Free Radic. Biol. Med..

[19] DiMarco T., Giulivi C. (2007). Current analytical methods for the detection of dityrosine, a biomarker of oxidative stress, in biological samples. Mass Spectrom. Rev..

[20] Fukuchi Y., Miura Y., Nabeno Y., Kato Y., Osawa T., Naito M. (2008). Immunohistochemical detection of oxidative stress biomarkers, dityrosine and N(epsilon)-(hexanoyl)lysine, and C-reactive protein in rabbit atherosclerotic lesions. J. Atheroscler. Thromb..

[21] Colombo G., Reggiani F., Cucchiari D., Portinaro N.M., Giustarini D., Rossi R., Garavaglia M.L., Saino N., Milzani A., Badalamenti S. (2017). Plasma protein-bound di-tyrosines as biomarkers of oxidative stress in end stage renal disease patients on maintenance haemodialysis. BBA Clin..

[22] Il’yasova D., Scarbrough P., Spasojevic I. (2012). Urinary biomarkers of oxidative status. Clin. Chim. Acta.

[23] Al-Hilaly Y.K., Williams T.L., Stewart-Parker M., Ford L., Skaria E., Cole M., Bucher W.G., Morris K.L., Sada A.A., Thorpe J.R. (2013). A central role for dityrosine crosslinking of Amyloid-beta in Alzheimer’s disease. Acta Neuropathol. Commun..

[24] Al-Hilaly Y.K., Biasetti L., Blakeman B.J., Pollack S.J., Zibaee S., Abdul-Sada A., Thorpe J.R., Xue W.F., Serpell L.C. (2016). The involvement of dityrosine crosslinking in alpha-synuclein assembly and deposition in Lewy Bodies in Parkinson’s disease. Sci. Rep..

[25] Mannino M.H., Patel R.S., Eccardt A.M., Perez Magnelli R.A., Robinson C.L.C., Janowiak B.E., Warren D.E., Fisher J.S. (2019). Myoglobin as a versatile peroxidase: Implications for a more important role for vertebrate striated muscle in antioxidant defense. Comp. Biochem. Physiol. Part B.

[26] La Mar G., Satterlee J., De Ropp J., Kadish K., Smith K., Guilard R. (2000). Nuclear magnetic resonance of hemoproteins. The Porphrylin Handbook.

[27] Yamamoto Y. (1998). NMR study of active sites in paramagnetic haemoproteins. Annu. Rep. NMR Spectrosc..

[28] Luthje S., Meisrimler C.N., Hopff D., Schutze T., Koppe J., Heino K. (2014). Class III peroxidases. Methods Mol. Biol..

[29] Shevchenko A., Wilm M., Vorm O., Mann M. (1996). Mass spectrometric sequencing of proteins silver-stained polyacrylamide gels. Anal. Chem..

[30] Basu S., Kirley T.L. (2005). Identification of a tyrosine residue responsible for N-acetylimidazole-induced increase of activity of ecto-nucleoside triphosphate diphosphohydrolase 3. Purinergic Signal..

[31] Lin Y.W. (2015). The broad diversity of heme-protein cross-links: An overview. Biochim. Biophys. Acta.

[32] Silaghi-Dumitrescu R., Reeder B.J., Nicholls P., Cooper C.E., Wilson M.T. (2007). Ferryl haem protonation gates peroxidatic reactivity in globins. Biochem. J..

[33] DeGray J.A., Gunther M.R., Tschirret-Guth R., Ortiz de Montellano P.R., Mason R.P. (1997). Peroxidation of a specific tryptophan of metmyoglobin by hydrogen peroxide. J. Biol. Chem..

[34] Kroger-Ohlsen M.V., Ostdal H., Andersen M.L. (2003). The effect of pH on the oxidation of bovine serum albumin by hypervalent myoglobin species. Arch. Biochem. Biophys..

[35] Mitsos S.E., Kim D., Lucchesi B.R., Fantone J.C. (1988). Modulation of myoglobin-H2O2-mediated peroxidation reactions by sulfhydryl compounds. Lab. Investig..

[36] Reeder B.J., Svistunenko D.A., Sharpe M.A., Wilson M.T. (2002). Characteristics and mechanism of formation of peroxide-induced heme to protein cross-linking in myoglobin. Biochemistry.

[37] Detweiler C.D., Lardinois O.M., Deterding L.J., de Montellano P.R., Tomer K.B., Mason R.P. (2005). Identification of the myoglobin tyrosyl radical by immuno-spin trapping and its dimerization. Free Radic. Biol. Med..

[38] Svistunenko D.A., Dunne J., Fryer M., Nicholls P., Reeder B.J., Wilson M.T., Bigotti M.G., Cutruzzola F., Cooper C.E. (2002). Comparative study of tyrosine radicals in hemoglobin and myoglobins treated with hydrogen peroxide. Biophys. J..

[39] Richardson R.S., Noyszewski E.A., Kendrick K.F., Leigh J.S., Wagner P.D. (1995). Myoglobin O2 desaturation during exercise. Evidence of limited O2 transport. J. Clin. Investig..

[40] Richardson R.S., Newcomer S.C., Noyszewski E.A. (2001). Skeletal muscle intracellular PO(2) assessed by myoglobin desaturation: Response to graded exercise. J. Appl. Physiol..

[41] Sakellariou G.K., Vasilaki A., Palomero J., Kayani A., Zibrik L., McArdle A., Jackson M.J. (2013). Studies of mitochondrial and nonmitochondrial sources implicate nicotinamide adenine dinucleotide phosphate oxidase(s) in the increased skeletal muscle superoxide generation that occurs during contractile activity. Antioxid. Redox Signal..

[42] Rainey W.T.J., McDufie H.F., Hess D.N., Yeatts L.B. (1964). Kinetics of the Thermal Decomposition of Biphenyl.

[43] Paviani V., Queiroz R.F., Marques E.F., Di Mascio P., Augusto O. (2015). Production of lysozyme and lysozyme-superoxide dismutase dimers bound by a ditryptophan cross-link in carbonate radical-treated lysozyme. Free Radic. Biol. Med..

[44] Leinisch F., Mariotti M., Rykaer M., Lopez-Alarcon C., Hagglund P., Davies M.J. (2017). Peroxyl radical- and photo-oxidation of glucose 6-phosphate dehydrogenase generates cross-links and functional changes via oxidation of tyrosine and tryptophan residues. Free Radic. Biol. Med..

[45] Leo G., Altucci C., Bourgoin-Voillard S., Gravagnuolo A.M., Esposito R., Marino G., Costello C.E., Velotta R., Birolo L. (2013). Ultraviolet laser-induced cross-linking in peptides. Rapid Commun. Mass Spectrom..

[46] Osapay K., Tran D., Ladokhin A.S., White S.H., Henschen A.H., Selsted M.E. (2000). Formation and characterization of a single Trp-Trp cross-link in indolicidin that confers protease stability without altering antimicrobial activity. J. Biol. Chem..

[47] Paviani V., Galdino G.T., dos Prazeres J.N., Queiroz R.F., Augusto O. (2018). Ditryptophan cross-links as novel products of protein oxidation. J. Braz. Chem. Soc..

[48] Giulivi C., Romero F.J., Cadenas E. (1992). The interaction of Trolox C, a water-soluble vitamin E analog, with ferrylmyoglobin: Reduction of the oxoferryl moiety. Arch. Biochem. Biophys..

[49] Aguirre E., Rodriguez-Juarez F., Bellelli A., Gnaiger E., Cadenas S. (2010). Kinetic model of the inhibition of respiration by endogenous nitric oxide in intact cells. Biochim. Biophys. Acta.

[50] Madej T., Lanczycki C.J., Zhang D., Thiessen P.A., Geer R.C., Marchler-Bauer A., Bryant S.H. (2014). MMDB and VAST+: Tracking structural similarities between macromolecular complexes. Nucleic Acids Res..

[51] Wang C.S., Pan H., Weerasekare G.M., Stewart R.J. (2015). Peroxidase-catalysed interfacial adhesion of aquatic caddisworm silk. J. R. Soc. Interface.

[52] Raven D.J., Earland C., Little M. (1971). Occurrence of dityrosine in Tussah silk fibroin and keratin. Biochim. Biophys. Acta.

[53] LaBella F., Keeley F., Vivian S., Thornhill D. (1967). Evidence for dityrosine in elastin. Biochem. Biophys. Res. Commun..

[54] Foerder C.A., Shapiro B.M. (1977). Release of ovoperoxidase from sea urchin eggs hardens the fertilization membrane with tyrosine crosslinks. Proc. Natl. Acad. Sci. USA.

[55] Kato Y., Maruyama W., Naoi M., Hashizume Y., Osawa T. (1998). Immunohistochemical detection of dityrosine in lipofuscin pigments in the aged human brain. FEBS Lett..

[56] Mayer F., Falk M., Huhn R., Behmenburg F., Ritz-Timme S. (2016). Dityrosine as a marker of acute myocardial infarction? Experiments with the isolated Langendorff heart. Int. J. Leg. Med..

[57] Atwood C.S., Perry G., Zeng H., Kato Y., Jones W.D., Ling K.Q., Huang X., Moir R.D., Wang D., Sayre L.M. (2004). Copper mediates dityrosine cross-linking of Alzheimer’s amyloid-beta. Biochemistry.

[58] Khan A.A., Mao X.O., Banwait S., Jin K., Greenberg D.A. (2007). Neuroglobin attenuates beta-amyloid neurotoxicity in vitro and transgenic Alzheimer phenotype in vivo. Proc. Natl. Acad. Sci. USA.

[59] Szymanski M., Wang R., Fallin M.D., Bassett S.S., Avramopoulos D. (2010). Neuroglobin and Alzheimer’s dementia: Genetic association and gene expression changes. Neurobiol. Aging.

[60] Reeder B.J., Cutruzzola F., Bigotti M.G., Hider R.C., Wilson M.T. (2008). Tyrosine as a redox-active center in electron transfer to ferryl heme in globins. Free Radic. Biol. Med..

[61] Reeder B.J., Wilson M.T. (2001). The effects of pH on the mechanism of hydrogen peroxide and lipid hydroperoxide consumption by myoglobin: A role for the protonated ferryl species. Free Radic. Biol. Med..

[62] Weller P.A., Price M., Isenberg H., Edwards Y.H., Jeffreys A.J. (1986). Myoglobin expression: Early induction and subsequent modulation of myoglobin and myoglobin mRNA during myogenesis. Mol. Cell. Biol..

[63] Moller P., Sylven C. (1981). Myoglobin in human skeletal muscle. Scand. J. Clin. Lab. Investig..

[64] Kreutzer U., Jue T. (2004). Role of myoglobin as a scavenger of cellular NO in myocardium. Am. J. Physiol. Heart Circ. Physiol..

[65] Gussakovsky E., Yang Y., Rendell J., Jilkina O., Kupriyanov V. (2010). Mapping the myoglobin concentration, oxygenation, and optical pathlength in heart ex vivo using near-infrared imaging. Anal. Biochem..

[66] Swartling J., Pålsson S., Platonov P., Olsson S.B., Andersson-Engels S. (2003). Changes in tissue optical properties due to radio-frequency ablation of myocardium. Med. Biol. Eng. Comput..

[67] Mason S.A., Baptista R., Della Gatta P.A., Yousif A., Russell A.P., Wadley G.D. (2014). High-dose vitamin C supplementation increases skeletal muscle vitamin C concentration and SVCT2 transporter expression but does not alter redox status in healthy males. Free Radic. Biol. Med..

[68] Jackson M.J. (2009). Redox regulation of adaptive responses in skeletal muscle to contractile activity. Free Radic. Biol. Med..

[69] Palomero J., Pye D., Kabayo T., Spiller D.G., Jackson M.J. (2008). In situ detection and measurement of intracellular reactive oxygen species in single isolated mature skeletal muscle fibers by real time fluorescence microscopy. Antioxid. Redox Signal..

[70] Akl M.G., Fawzy E., Deif M., Farouk A., Elshorbagy A.K. (2017). Perturbed adipose tissue hydrogen peroxide metabolism in centrally obese men: Association with insulin resistance. PLoS ONE.

